# Nimodipine vs. Milrinone – Equal or Complementary Use? A Retrospective Analysis

**DOI:** 10.3389/fneur.2022.939015

**Published:** 2022-07-14

**Authors:** Jennifer Jentzsch, Svitlana Ziganshyna, Dirk Lindner, Helena Merkel, Simone Mucha, Stefan Schob, Ulf Quäschling, Karl-Titus Hoffmann, Robert Werdehausen, Dirk Halama, Khaled Gaber, Cindy Richter

**Affiliations:** ^1^Department of Neuroradiology, Leipzig University Hospital, Leipzig, Germany; ^2^Transplant Coordinator Unit, Leipzig University Hospital, Leipzig, Germany; ^3^Department of Neurosurgery, Leipzig University Hospital, Leipzig, Germany; ^4^Department of Radiology, Halle University Hospital, Halle, Germany; ^5^Department of Radiology, Kantonsspital Baselland, Liestal, Switzerland; ^6^Department of Anaesthesiology and Intensive Care Medicine, Leipzig University Hospital, Leipzig, Germany; ^7^Department of Oral and Maxillofacial Surgery, Leipzig University Hospital, Leipzig, Germany

**Keywords:** delayed cerebral ischemia, endovascular procedures, milrinone, nimodipine, subarachnoid hemorrhage, cerebral vasospasm

## Abstract

**Background:**

Cerebral vasospasm (CVS) continues to account for high morbidity and mortality in patients surviving the initial aneurysmal subarachnoid hemorrhage (SAH). Nimodipine is the only drug known to reduce delayed cerebral ischemia (DCI), but it is believed not to affect large vessel CVS. Milrinone has emerged as a promising option. Our retrospective study focused on the effectiveness of the intra-arterial application of both drugs in monotherapy and combined therapy.

**Methods:**

We searched for patients with aneurysmal SAH, angiographically confirmed CVS, and at least one intra-arterial pharmacological angioplasty. Ten defined vessel sections on angiograms were assessed before and after vasodilator infusion. The improvement in vessel diameters was compared to the frequency of DCI-related cerebral infarction before hospital discharge and functional outcome reported as the modified Rankin Scale (mRS) score after 6 months.

**Results:**

Between 2014 and 2021, 132 intra-arterial interventions (144 vascular territories, 12 bilaterally) in 30 patients were analyzed for this study. The vasodilating effect of nimodipine was superior to milrinone in all intradural segments. There was no significant intergroup difference concerning outcome in mRS (*p* = 0.217). Only nimodipine or the combined approach could prevent DCI-related infarction (both 57.1%), not milrinone alone (87.5%). Both drugs induced a doubled vasopressor demand due to blood pressure decrease, but milrinone alone induced tachycardia.

**Conclusions:**

The monotherapy with intra-arterial nimodipine was superior to milrinone. Nimodipine and milrinone may be used complementary in an escalation scheme with the administration of nimodipine first, complemented by milrinone in cases of severe CVS. Milrinone monotherapy is not recommended.

## Introduction

Subarachnoid hemorrhage (SAH) is in about 85% of cases caused by cerebral aneurysm rupture (1). A distinct feature of SAH is the relatively young age of the affected patients. The peak age of incidence lies between 40 and 60 years. Despite recent advances in diagnostic and therapeutic possibilities, the 30-day mortality of aneurysmal SAH exceeds 30% (2), with the occurrence and management of vasospasms being an important factor (3). The survival rate is reported at 50%. Among survivors of SAH, up to 40% cannot return to their previous occupation, while 44–93% of patients need some form of assistance with daily living activities like finances or housekeeping (2). Thus, new therapeutic strategies for the treatment of SAH and subsequent management of vasospasms are urgently required to improve patients' outcomes.

Dysregulation of the cerebral vasculature, mainly at the larger extraparenchymal vessels, remains a target for endovascular treatment and improves the outcome of patients with severe, refractory vasospasms (1). Vasospasm occurs in up to 70% of angiograms after SAH (3). It is defined as a self-limited narrowing of the vessels, with a typical onset 3 to 5 days after hemorrhage, maximal narrowing at 5 to 14 days, and gradual resolution over 2 to 4 weeks.

Nimodipine and milrinone are the most applied drugs for intra-arterial spasmolysis. Retrospective studies have found large variability in dosing, route, and cointerventions (4–7). Both drugs influence the amount of cytosolic calcium in different manners. Nimodipine, a calcium channel antagonist, reduces calcium influx into all smooth-muscle cells in the vessel wall by blocking the calcium channels (8). Milrinone is a selective phosphodiesterase (PDE) 3 inhibitor which augments myocardial contractility, relaxes vascular smooth muscle, and inhibits platelet aggregation (9). These complementary mechanisms of action suggest the complementary use of nimodipine and milrinone.

In this retrospective study, the aim was to analyze the effects of nimodipine, milrinone, and the combined use of both agents on different vessel segments to define the criteria for their application. We hypothesized that milrinone could be as effective as nimodipine for improving arterial vessel diameters and that a multiple-vasodilator regimen may provide a greater benefit in angiographic and clinical outcomes than monotherapy.

## Materials and Methods

### Population/Study Design and Recruitment

We screened our hospital records for patients admitted to our clinic after SAH between January 2014 and July 2021 with aneurysmal SAH, angiographically confirmed CVS, and at least one intra-arterial pharmacological angioplasty. The local Ethics Committee of our Medical Faculty approved this study (reference number: 412/19-ek). Inclusion criteria were: (1) an aneurysmatic SAH verified by computer tomography (CT), magnetic resonance imaging (MRI), or lumbar puncture, and (2) pharmacological intra-arterial spasmolysis. Patients with mycotic or traumatic-induced pseudoaneurysms and aneurysms associated with arteriovenous malformations were excluded.

Delayed cerebral ischemia (DCI) is the recommended term to describe the post-SAH delayed clinical deterioration after other potential identifiable causes have been ruled out. DCI can lead to infarction. To identify DCI-related infarction, available non-contrast CT at hospitalization, after aneurysm treatment, at the onset of vasospasm before endovascular treatment, and after the vasospasm phase before discharge were analyzed for signs of infarction. Infarctions within 48 h after aneurysm treatment and up to 3 days after initial SAH were assessed as SAH-related or treatment-associated complications. New infarctions up to 21 days after SAH due to other potential causes (f.e. EVD, herniation, ICH, periprocedural complication) have been ruled out.

After 6 months, all neurosurgical patients were routinely summoned to our clinic to evaluate the functional outcome. The modified Ranking Scale (mRS) was subsequently assessed based on the medical report.

### Standard of Care After SAH

All patients were monitored in our neurointensive care unit (Supplement 1). Nimodipine (Hexal AG, Sandoz International GmbH, Holzkirchen, Germany) was given orally or *via* gastric tube (60 mg every 4 h) from admission until day 21 after SAH. The standard of care compromised normovolemia and normoglycemia. The mean arterial blood pressure was always sustained above 75 to 85 mmHg to support a cerebral perfusion pressure (CPP) over 65 mmHg. The blood pressure target depended additionally on patient age, pre-existing hypertension, and cardiac function. The intravenous administration of the vasopressor norepinephrine or the vasodilator urapidil was used for blood pressure management. To prevent thrombosis and/or pulmonary embolism, all patients received low molecular weight heparin in a prophylactic dosage (tinzaparin 4,500 IU/day). Corticosteroids or statins were not given routinely.

Monitoring included daily transcranial Doppler (TCD) investigation of cerebral arteries for 2 weeks following SAH. If cerebral vasospasm was suspected based on new neurological impairment or increased flow velocity in TCD above 120 cm/s in MCA or doubling within 24 h, CT was performed to detect new territorial infarctions. Intubated and sedated patients without a sufficient window for ultrasound assessment received a control CT after 7 days. CT perfusion analysis was additionally performed in cases with new infarctions to determine if further treatment is needed. Before indicating rescue therapy, there was always an interdisciplinary discussion between intensivists, neurosurgeons, and neuroradiologists. The potential patient's benefit had to exceed the risk of blood pressure drops and physical stress during transport. Lacunar or small territorial infarctions were not considered a contraindication for intra-arterial spasmolysis, as long as there was still a large vessel territory at risk. The initial DSA on admission served as a reference image to assess the severity of CVS.

### Vasodilator Infusion

Diagnostic DSA was performed using either a biplane Siemens system (Axiom Artis, Erlangen, Germany), a biplane Philips system (AlluraClarity, Philips Healthcare, Best, Netherlands), or a monoplane GE system (Innova 4100; GE Healthcare, Waukesha, USA). Iopromide (60–120 ml, containing 300 mg iodine per ml) was used as the contrast medium.

We regularly performed intra-arterial spasmolysis over the entire period, using nimodipine (10 mg/50 ml, Carinopharm, Elze, Germany), milrinone (Corotrop®, 10 mg/10 ml, Sanofi-Aventis, Frankfurt am Main, Germany), or a combination of both agents. In this retrospective study, patients were not randomized for one treatment. There was a staff turnover causing a serial use of nimodipine from 2014 to 2017 and milrinone from 2018 to 2021. The latter interventionalists also used a combination of both agents (2018–2021), especially in more severe cases.

Vasodilators were administered intra-arterial *via* 4-F or 5-F angiography catheters (Tempo® Catheter, Cordis, Miami, USA) with the same inner diameter. The femoral sheath and the catheter were continuously flushed with an infusion of heparin (1,000 U/L) and saline solution (0.9%) to prevent periprocedural thromboembolism. The patients' hemisphere most affected by vasospasm was defined as the main side. Vessel diameters were analyzed at the time when the dilating agent was directly admitted to the corresponding hemisphere. Nimodipine was applied with a dosage of 1.5 mg per vascular territory and a maximum of 3 mg per treatment session. As a rare exception, 6 mg in a single treatment was administered. When used separately, milrinone was applied with a dosage of 10 mg per vascular territory and a maximum of 20 mg per treatment. In rare cases, a maximum of 40 mg was used. Different agents were administered consecutively, typically over 30 −120 min. According to the criteria mentioned above, the treatment was repeated after 12 to 24 h or intermittently on demand when recurrence of vasospasm was suspected by TCD in unconscious patients or worsening of clinical symptoms. Continuous spasmolysis with indwelling microcatheters was not performed.

Since nimodipine and milrinone are vasodilative, norepinephrine (Arterenol®, Sanofi-Aventis, Frankfurt am Main, Germany with a dilution of 5,000 μg/50 ml was used as a vasopressor to compensate for drops in systemic blood pressure. The vasopressor demand, the mean arterial pressure (MAP), and the heart rate (HR) were assessed from the digital patient records 1 h before intra-arterial spasmolysis and during spasmolysis. The minimum MAP during spasmolysis was evaluated. Milrinone is known to increase the HR (10); therefore, the maximum HR during spasmolysis was analyzed.

### Angiographic Measurements

Only angiographic procedures with a direct intra-arterial infusion of vasodilating agents in the internal carotid artery were evaluated.

**Figure 1:**
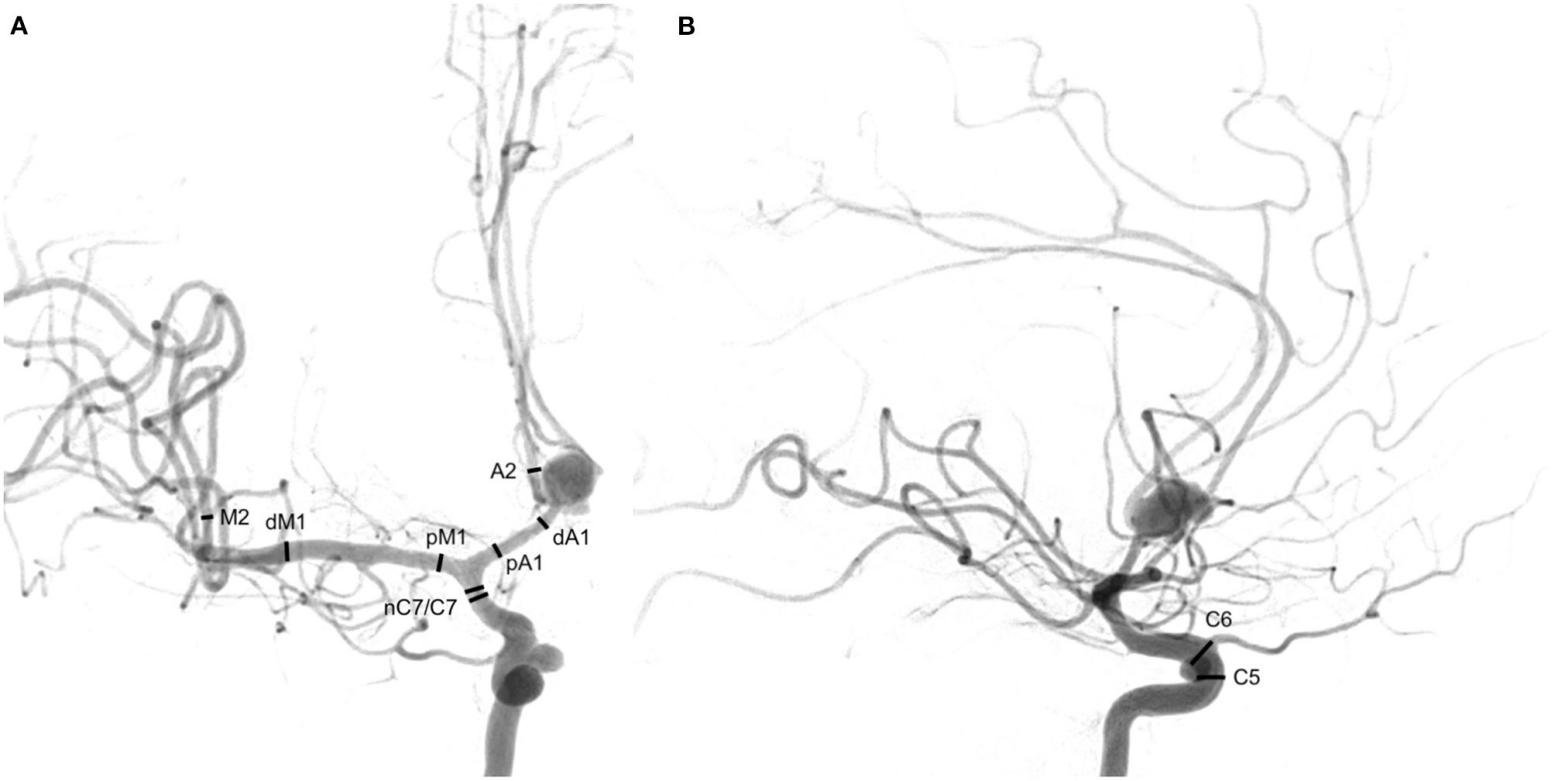
Ten measured points. **(A)** Posterior-anterior projection of an internal carotid artery angiogram, *C7* terminal segment of the internal carotid artery, *nC7* narrowest point of C7, *pM1* proximal horizontal segment of the middle cerebral artery, *dM1* distal horizontal segment of the middle cerebral artery, *M2* insular segment of the middle cerebral artery, *pA1* proximal pre-communication segment of the anterior cerebral artery, *dA1* distal pre-communication segment of the anterior cerebral artery, *A2* post-communicating segment of the anterior cerebral artery. **(B)** Lateral projection of an internal carotid artery angiogram, *C5* clinoid segment of the internal carotid artery, *C6* ophthalmic segment of the internal carotid artery. In lateral projection: C5: 2 mm proximal to the origin of the ophthalmic artery and C6: at the origin of the ophthalmic artery. In posterior-anterior projection: C7: 2 mm proximal to the carotid T, nC7: the narrowest location in vasospastic arteries of C7 segments, pM1: 2 mm distal to carotid T, dM1: 2 mm proximal to middle cerebral artery bifurcation, M2: 4 mm distal to the M1/M2 transition, pA1: 2 mm distal to carotid T, dA1: 2 mm proximal to change of course and A2: 2 mm distal to change of course.

Vessel diameters before infusion (pre-infusion diameter = PrID) and after completion of pharmacological treatment (post-infusion diameter = PoID) were measured at ten defined vessel segments: segment C5 of the extradural internal carotid artery (ICA), segment C6 and C7 of the intradural ICA, the narrowest point of C7 (nC7), the proximal M1 segment (pM1) the distal M1 segment (dM1) and the proximal M2 segment (M2) of the middle cerebral artery, the proximal A1 segment (pA1), the distal A1 segment (dA1) and the proximal A2 segment (A2) of the anterior cerebral artery. The drug-specific spasmolytic effect was defined by calculating the difference of PrID and PoID for each measured point. The degree of angiographic response was defined by the improvement percentage ratio (IPR) calculated as IPR = (PoID/PrID−1) x 100%. All values were measured on a diagnostic workstation (syngo.plaza, VB30C, Siemens Health Care, Erlangen, Germany) according to a defined protocol (Figure 1).

The investigator was blinded to the patients' treatment and retrospectively decided whether vessel narrowing was presumably due to a spasm or a pre-existing condition, such as developmental hypoplasia or atherosclerosis. The proximal ACA was considered hypoplastic if the contralateral A1-segment and the AComA were large and the A2-segments were well filled. Confidence in identifying A1 hypoplasia was increased if no spasm was present in the ipsilateral A2 segment. In the case of the proximal bifurcation variant of the MCA, the most prominent branch of the M1 segment was evaluated. The two most prominent M2 branches were measured if there was a trifurcation or quattrofurcation.

The values were not measured at the site of aneurysms or implanted materials e.g., coils. Vessel segments with stents of any kind were excluded.

### Statistical Analysis

Statistical analyses were performed with SPSS version 27.0 (IBM Corporation, New York, USA) and R version 4.0.1 (The R Foundation for Statistical Computing, Vienna, Austria). Data were first analyzed by the Shapiro-Wilk test for normal distribution. Subsequently, the *t*-test and chi-square test were used as appropriate. A two-tailed value of *p* < 0.05 was considered indicative of statistical significance.

## Results

We identified 30 patients with 129 intra-arterial spasmolysis in 144 vascular territories (12 bilateral) due to cerebral vasospasm (Table 1). Twenty-three patients underwent coiling (days to treatment after SAH: median: 0 days, range: 0–7 days), four patients clipping (days to treatment after SAH: median: 0 days, range: 0–9 days), and two patients with more than one aneurysm received a combined treatment of coiling and clipping (days to treatment after SAH median: 0.5 days, range: 0–9 days). In one case, no source of bleeding could be found.

**Table 1:** Baseline demographics.

	**Nimodipine**	**Milrinone**	**Nimodipine and Milrinone**
**General demographic data**			
Patients (*n*)	15	8	7
Female (%)	8 (53)	6 (75)	6 (86)
Mean age (range)	53 (28–76)	47 (32–77)	47 (31–62)
**Fisher score (mean** **±SEM)** II (%) III (%) IV (%)	3.5 ± 0.6 1 (6.6) 5 (33.3) 9 (60.0)	3.6 ± 0.7 1 (12.5) 1 (12.5) 6 (75.0)	4.0 ± 0.0 0 (0.0) 0 (0.0) 7 (100.0)
**WFNS score (mean** **±SEM)** I (%) II (%) III (%) IV (%) V (%)	2.8 ± 1.9 7 (46.7) 1 (6.6) 0 (0.0) 2 (13.3) 5 (33.3)	2.3 ± 1.8 5 (62.5) 0 (0.0) 0 (0.0) 2 (25.0) 1 (12.5)	3.0 ± 1.4 2 (25.0) 0 (0.0) 1 (12.5) 4 (50.0) 0 (0.0)
**Hunt & Hess score (mean** **±SEM)** I (%) II (%) III (%) IV (%) V (%)	3.3 ± 1.4 1 (6.6) 4 (26.7) 4 (26.7) 1 (6.6) 5 (33.3)	3.1 ± 1.1 0 (0.0) 3 (37.5) 2 (25.0) 2 (25.0) 1 (12.5)	3.1 ± 0.7 0 (0.0) 1 (12.5) 4 (50.0) 2 (25.0) 0 (0.0)
**Treatment**			
EVD (%)	10 (66.6)	8 (100.0)	7 (100.0)
ETA (%)	9 (60.0)	7 (87.5)	7 (100.0)
Endovascular treatment (%)	14 (93.3)	8 (100.0)	4 (57.1)
Clipping (%)	2 (13.3)	0 (0.0)	4 (57.1)

*Our results are based on the analysis of vessel diameters of 30 patients suffering from protracted cerebral vasospasm admitted to our hospital. The clinical data of each group are summarized according to the applied spasmolytic agents. ETA, endotracheal anesthesia; EVD, external ventricular drainage; SEM, standard error of the mean; WFNS, World Federation of Neurosurgical Societies*.

### General Demographic Data

Regarding clinical scores, patients who received monotherapy with nimodipine or milrinone did not relevant differ in Fisher score, Hunt & Hess score, or WFNS score on admission (Table 1). Patients who received combined therapy with milrinone and nimodipine were predominantly female (86%). These patients were seriously injured before the onset of CVS, with overrepresented intracranial hemorrhages and a worse Fisher score (*n* = 4), leading to a higher proportion of clipped aneurysms in these patients (*n* = 4). This is partly caused by a profound selection bias that has to be kept in mind when interpreting outcome data. Therefore, a prospective design is needed to refer valuable clinical outcome data to pharmacological intra-arterial CVS treatment. Intubation and external ventricular drainage (EVD) were less frequent in patients treated with nimodipine only compared to the other patients.

### Periprocedural Complications Related to Aneurysm Treatment

After endovascular aneurysm treatment, post-therapeutic infarctions occurred in three instances, and intracranial hemorrhage was observed once (*n* = 4, WFNS score: 3, HH score: 3, Fisher score: 3.75). After clipping, three patients developed post-therapeutic infarctions (*n* = 3, WFNS score: 3, HH score: 3.67, Fisher score: 4). Two patients with multiple aneurysms and combined treatment revealed an infarction or an intracranial hemorrhage (ICH) due to aneurysm rupture during clipping (*n* = 2, WFNS score: 4.5, HH score: 4, Fisher score: 4).

### Intra-arterial Spasmolysis and Angiographic Effect

Reasons to perform intra-arterial spasmolysis are summarized in Table 2. A critical increase above 120 cm/s in TCD of the MCA in fourteen patients indicated cerebral vasospasm. Eleven patients showed clinical worsening as initial symptoms. CT perfusion analysis in 21 cases (70%) revealed perfusion deficits in 13 cases and new territorial infarctions were already seen in five patients, supporting the diagnosis of vasospasm. All patients underwent digital subtraction angiography (DSA) to confirm, assess and treat vasospasm. The first intra-arterial spasmolysis was performed on average on day 9 (day 3–21).

**Table 2:** Intra-arterial spasmolysis and endpoint analysis.

	**Nimodipine**	**Milrinone**	**Nimodipine and Milrinone**
Patients (*n*)	15	8	7
Number of repeated interventions per patient (mean, range)	3.9 (1–14)	4.4 (1–9)	5.6 (1–10)
**Reasons to perform intra-arterial spasmolysis**			
Altered level of consciousness or new neurologic deficit (%)	7 (46.7)	3 (37.5)	1 (14.3)
New infarction on CT (%)	2 (13.3)	2 (25.0)	1 (14.3)
Perfusion impairment on CT (%)	7 (46.7)	4 (50.0)	2 (28.6)
TCD velocity increase (%)	5 (33.3)	3 (37.5)	6 (16.7)
Intubated patients (%)	9 (60.0)	7 (87.5)	7 (100.0)
**Circulatory parameters before and during i.a. spasmolysis**			
MAP: 1 h before (mmHg, mean, range)	88.2 (66–116)	90.5 (65–116)	95.9 (72–121)
MAP: minimum (mmHg, mean, range)	78.9 (57–103)	84.2 (45–118)	88.0 (48–117)
MAP: *p*-values	0.000***	0.042*	0.106
HR: 1 h before (bpm, mean, range)	74.8 (51–131)	80.0 (57–105)	77.6 (57–113)
HR: maximum (bpm, mean, range)	78.4 (55–124)	100.7 (59–135)	92.5 (66–137)
HR: *p*-values	0.244	0.000***	0.006**
Vasopressor demand: 1 h before (flow rate in μg/kg/min, mean, range)	0.074	0.593	0.036
Vasopressor demand: maximum (flow rate in μg/kg/min, mean, range)	0.155	0.172	0.134
**Outcome**			
Overall (mean ± SEM) mRS 0 (%) mRS 1 (%) mRS 2 (%) mRS 3 (%) mRS 4 (%) mRS 5 (%) mRS 6 (%)	2.8 ± 0.5 1 (6.7) 6 (40.0) 1 (6.7) 0 (0.0) 2 (13.3) 4 (26.7) 1 (6.7)	3.4 ± 0.8 1 (12.5) 2 (25.0) 0 (0.0) 0 (0.0) 1 (12.5) 3 (37.5) 1 (12.5)	4.3 ± 0.6 0 (0.0) 1 (14.3) 0 (0.0) 0 (0.0) 2 (28.6) 3 (42.9) 1 (14.3)
DCI-related infarctions despite i.a. spasmolysis (%)	8 (53.3)	7 (87.5)	4 (57.1)

Circulatory data and vasopressor demand were retrospectively assessed from the digital patient records 1 h before intra-arterial spasmolysis and during spasmolysis. Norepinephrine was used as vasopressor with a dilution of 5,000 μg/50 ml. CT, computer tomography; DCI, delayed cerebral ischemia; HR, heart rate; i.a., intra-arterial; MAP, mean arterial pressure; mRS, modified Rankin Scale; SEM, standard error of the mean; TCD, transcranial doppler. *p-value < 0.05, significant; **p-value < 0.01, highly significant; ***p-value < 0.001, extremely significant.

We analyzed one hundred-forty-four episodes of spasmolytic therapies in 30 patients. The applied vasodilating agents included nimodipine (main = 48, contralateral = 5), milrinone (main = 33, contralateral = 14), or a combination of both (nimodipine plus milrinone, main = 19, contralateral = 10) summarized in Table 3. Twenty-three patients were intubated during spasmolysis, and seven were treated awake with anesthesiological stand-by.

**Table 3:** Drug-depended distribution of angiograms.

	**Nimodipine**		**Milrinone**		**Nimodipine** **+** **Milrinone (NM)**	
	**main**	**contralateral**	**main**	**contralateral**	**main**	**contralateral**
patients	14 (+ 2 NM)	2 (+ 2 NM)	8 (+ 5 NM)	4 (+ 3 NM)	7	4
angiograms	48 (+ 1 NM)	3 (+ 2 NM)	33 (+ 14 NM)	9 (+ 5 NM)	19	10

*The most affected side with drug application first was named the main side. The contralateral side was only evaluated if the spasmolytic agent was administered directly. Patients and angiograms in brackets with the supplement NM count among patients who received the combination of nimodipine and milrinone. Their vessel diameters were analyzed in the nimodipine or milrinone group if only one spasmolytic agent was applied in this treatment*.

**Figure 2:**
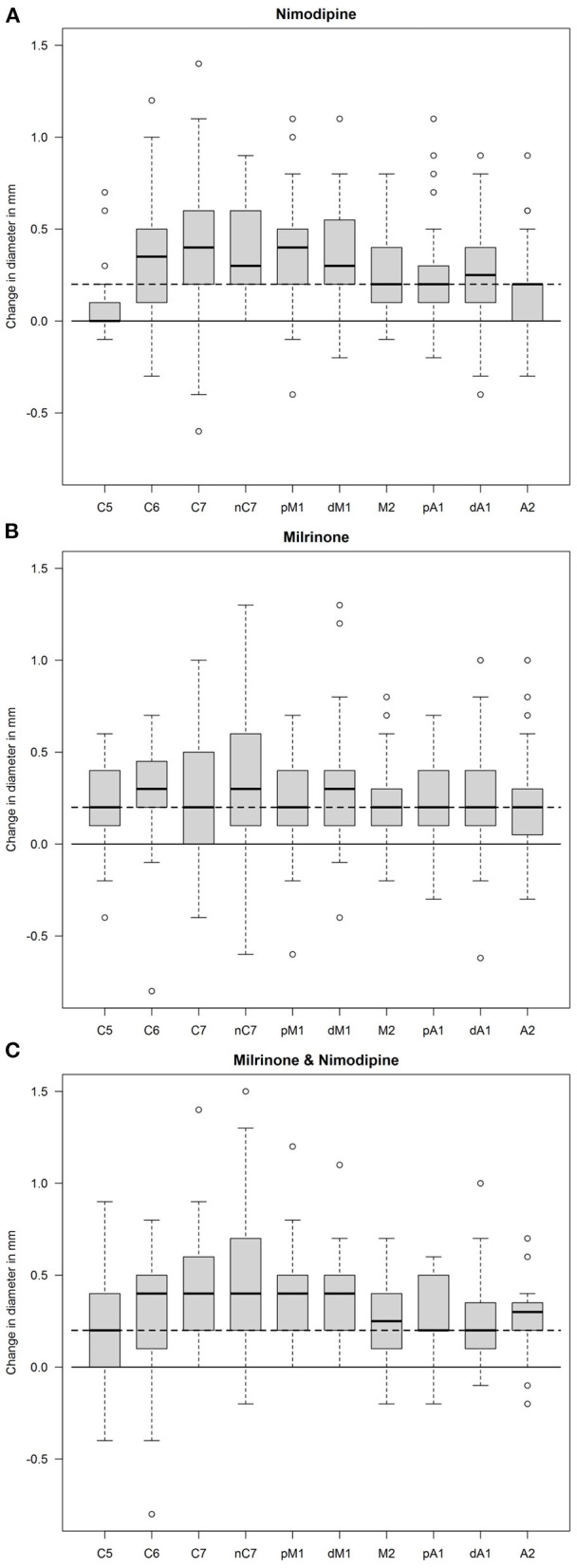
Box plot of diameter differences. The difference in vessel diameters (in mm) under pharmacological spasmolysis is depicted for each measured point (see Figure 1). Orientation lines in the neutral zone (continuous) and 0.2 mm difference (dashed) are given in each box plot. **(A)**: nimodipine, **(B)**: milrinone, **(C)**: nimodipine and milrinone.

The IPR for each measured point is depicted in boxblots for each group of drugs in Figure 2. *P*-values of the calculated difference of PrID and PoID for each drug group are shown in Table 4. Both agents had a highly significant vasodilating effect on all measured vessel segments, except the extradural C5 segment. Nimodipine exceeded in all intradural segments compared to milrinone. The intergroup comparison showed a highly significant *p*-value for the pM1 between nimodipine and milrinone. The extradural C5 segment responded higher to the milrinone application. The combined approach augmented the IPR in the C5, pA1, A2, and M2 segments compared to monotherapy with nimodipine, but significant differences were only detected for C5 and A2 segments.

**Table 4:** Comparison of spasmolytic effects.

**Vasodilator**	**IPR**			***p*** **-values: comparison of PrID and PoID**			***p*** **-values: intergroup comparison**		
	**Nimodipine (in %)**	**Milrinone (in %)**	**NM (in %)**	**Nimodipine**	**Milrinone**	**NM**	**Nimodipine vs. Milrinone**	**Nimodipine vs. NM**	**Milrinone vs. NM**
**Vessel segments**									
C5	2.1	5.9	5.6	0.757	0.090	0.215	0.012^*^	0.013^*^	0.756
C6	15.2	8.7	7.8	0.049^*^	0.045^*^	0.233	0.339	0.472	0.864
C7	22.1	15.6	20.6	0.000^***^	0.010^*^	0.000^***^	0.091	0.366	0.008^*^
nC7	25.5	22.3	25.5	0.001^**^	0.003^**^	0.000^***^	0.733	0.223	0.167
pM1	30.5	18.0	24.1	0.000^***^	0.002^**^	0.000^***^	0.008^*^	0.965	0.020^*^
dM1	36.7	24.3	25.3	0.000^***^	0.000^***^	0.011^*^	0.479	0.451	0.141
M2	30.9	22.6	36.1	0.000^***^	0.000^***^	0.030^*^	0.472	0.777	0.359
pA1	29.5	23.2	35.3	0.004^**^	0.012^*^	0.042^*^	0.267	0.964	0.326
dA1	53.7	31.8	29.7	0.000^***^	0.007^*^	0.041^*^	0.555	0.955	0.608
A2	24.3	23.9	29.0	0.040^*^	0.013^*^	0.001^***^	0.245	0.033^*^	0.284

*The table shows trichotomous from left to right the improvement percentage ratio (IPR), the p-values of the pre-infusion and post-infusion diameters, and the p-values of intergroup comparison of diameter differences for each segment separated into groups of spasmolytic agents. In most segments, nimodipine monotherapy displays the greatest IPR compared to milrinone monotherapy and the combined therapy of nimodipine and milrinone. P-values for the comparison of pre-infusion diameter (PrID) and post-infusion diameters (PoID) were significant, except for the extradural C5 segment in nimodipine monotherapy. The combined therapy of nimodipine and milrinone is superior to the monotherapy of milrinone in severe vasospasm, mainly affected vessel segments C7 and pM1. Nimodipine and milrinone led to significant intergroup differences in C5 and pM1 segments. Neither the patients receiving nimodipine nor those after combined treatment revealed an outstanding effect on vessel diameters. IPR, improvement percentage ratio; PoID, post-infusion diameter; PrID, pre-infusion diameter*.

* *p-value < 0.05, significant*;

** *p-value < 0.01, highly significant*;

*** *p-value < 0.001, extremely significant*.

### Undesired Effects of Intra-arterial Spasmolysis

There were no major periprocedural complications of intra-arterial spasmolysis, e.g., hemorrhage transformation of infarction, vessel occlusion, or dissection. Minor complications were femoral sheath dislocations or occlusions between the several endovascular treatments.

Circulatory data for MAP and HR 1 h before intra-arterial spasmolysis and during spasmolysis are specified in Table 2. The monotherapy with nimodipine or milrinone revealed a significant decrease in MAP, whereas the combined approach did not. The HR was highly significantly increased if milrinone was applied alone or in combination. The vasopressor demand (norepinephrine 5,000 μg/50 ml) doubled for both agents alone and tripled for the combined approach during endovascular treatment (Table 2). No vasopressor application was required in 14% (18/129) of treatments in 12 patients: 16.9% (10/59) treated with nimodipine, 10.2% (5/49) with milrinone, 14.3% (3/21) after receiving nimodipine and milrinone in combination.

One patient was susceptible to small dosages of milrinone (1 mg), resulting in a short-lasting tachyarrhythmia (<1 h). The spasmolytic treatment was escalated to nimodipine. Another patient showed rapid blood pressure drops to small milrinone dosages (<5 mg).

### DCI-Related Infarctions

**Figure 3:**
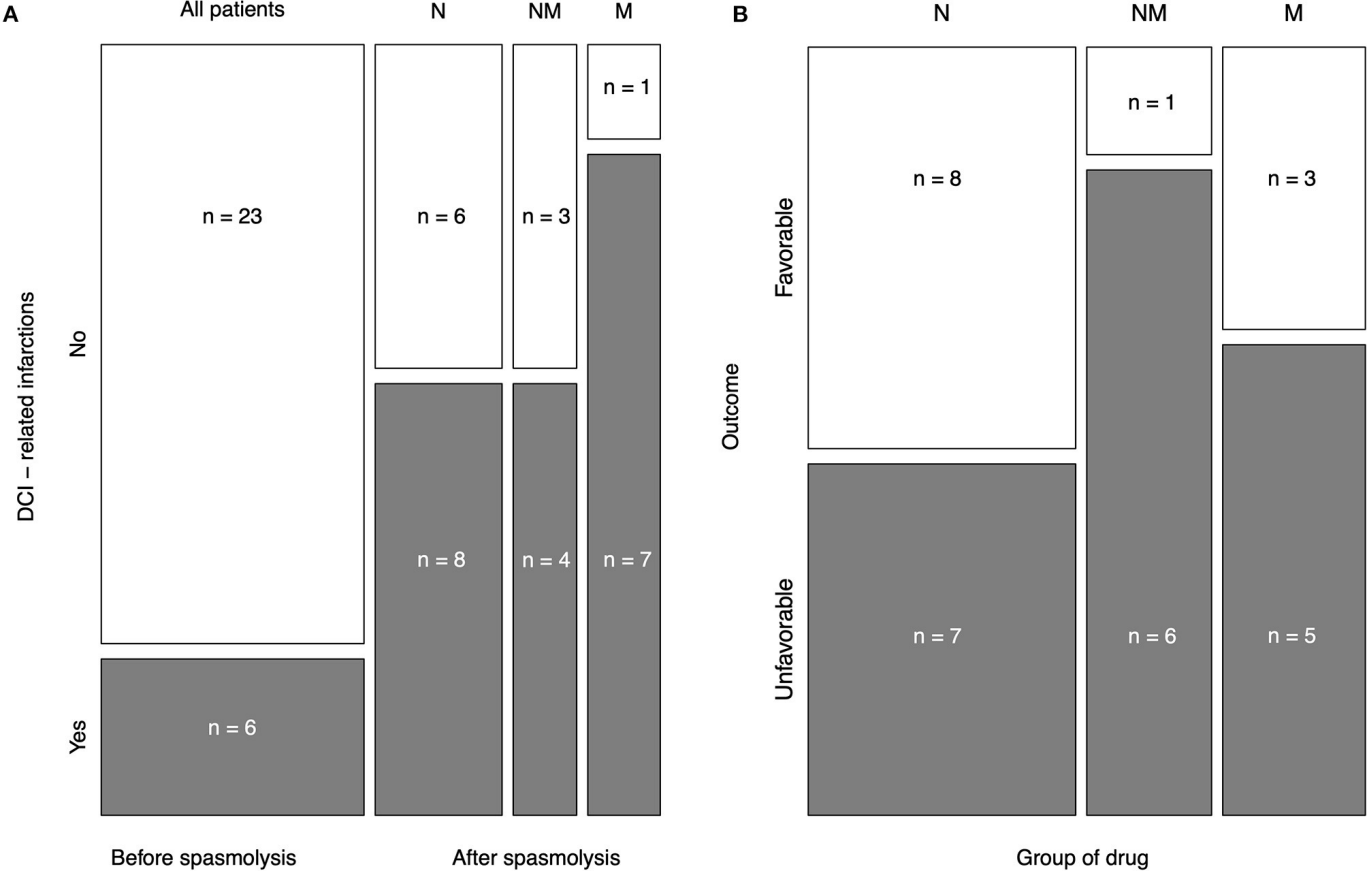
Mosaic plot of both endpoints. Delayed cerebral ischemia (DCI)-related infarctions and the outcome in modified Rankin Scale (mRS) score after 6 months were chosen as study endpoints. **(A)**: DCI-related infarction before and after intraarterial spasmolysis due to the applied spasmolytic agent is shown. One case was excluded for DCI analysis due to missing prespasmolytic CT. The minority of cases (20.7%) already experienced DCI before intra-arterial spasmolysis. Nimodipine and the combination of milrinone and nimodipine revealed the same proportion of DCI (57.1%). In most cases (87.5%), the monotherapy with intra-arterial milrinone could not prevent DCI-related infarctions. **(B)**: The outcome was subdivided into favorable (mRS 0–3) and unfavorable (mRS 4–6). Patients who received nimodipine only had the best outcome after 6 months assessed by mRS, followed by only milrinone-treated patients and patients after combined therapy. *N* = nimodipine, *M* = milrinone, *NM* = nimodipine and milrinone.

Six patients (20%) already suffered from DCI-related infarctions before intra-arterial spasmolysis (Figure 3). One patient was excluded for infarction analysis because of a missing CT scan before endovascular treatment. From the first intra-arterial spasmolysis until discharge, 19 patients (63.3%) developed additional DCI-related infarctions. Patients who received nimodipine or nimodipine plus milrinone had the same rate of DCI-related infarctions (57.1%). Patients who received milrinone only developed further postischemic infarctions in most cases (87.5%, Figure 3).

### Outcome

The outcome in mRS after 6 months is displayed in detail for each drug group in Table 2. Figure 3 displays the distribution of favorable (mRS 0–3) and unfavorable (mRS 4–6) outcomes according to the vasodilative agents for intra-arterial spasmolysis. Patients who received nimodipine only had the best overall outcome after 6 months assessed by mRS with 2.8 +/– 2.0 (0–6), followed by only milrinone-treated patients with 3.4 +/– 2.3 (0–6) and patients after combined therapy with 4.3 +/– 1.6 (Table 3). Figure 3 confirmed this distribution for favorable and unfavorable outcomes. The chi-square test for intergroup comparison did not reveal any significant difference (*p* = 0.216).

Three patients died of multi-infarct syndrome (*n* = 2) or general brain edema (*n* = 1). These patients had an initial Fisher score of 3 (*n* = 1) or 4 (*n* = 2). Patients who received the combination of nimodipine and milrinone had a worse clinical starting condition (Table 1). Still, the Fisher score (*p* = 0.145), WFNS (*p* = 0.261), or Hunt and Hess score (*p* =0.186) did not predict a significantly worse outcome in this study group.

## Discussion

Our data are retrospectively gathered from electronic files and images. In the last decade, neurosurgeons, intensivists, and neuroradiologists focused on cerebral vasospasm defined by angiography and TCD (4). Now-a-days, DCI is the recommended term to describe the post-SAH delayed clinical deterioration after other potential identifiable causes have been ruled out. Clinical features of DCI can be reversible or progress to cerebral infarction in the current definition of DCI. It is a multifactorial process involving endothelial injury with microspasm, blood-brain-barrier disruption, microthrombosis, cortical spreading depolarization, and loss of cerebral autoregulation, all predisposing the brain to further ischemic injury (1, 10). An angiographic resolution of intracranial proximal vessel vasospasm might not prevent DCI, but vessel diameters are a measurable indicator. In this retrospective study, cerebral injury following cerebral ischemia that occurred after SAH was called DCI-related infarction. Neurological condition was assessed only by means of analyzing the electronic files. Due to the retrospective design of this study, the authors did not assess DCI in a way suggested by Vergouwen et al. (11). Instead, this study was focused on comparing imaging findings. Tjerkstra et al. (4) showed in their nationwide, web-based survey that large variations remain in the definition, diagnosis, and treatment of DCI among intensivists, neurologists, and neurosurgeons. Many physicians still rely on angiography or TCD. For definition and diagnosis of vasospasm. Patients with severe SAH (Fisher 3–4) are frequently in a poor neurological condition (1), and the diagnosis of DCI, relying on clinical symptoms alone for a diagnosis of DCI according to Vergouwen et al. (11), seems suboptimal.

The decision algorithm of our hospital is based on worsening of neurological function or signs of CVS (TCD velocity increase) then leading to further imaging (native CT, CT perfusion, CT angiography). Patients with clinical worsening, vessel territories at risk, and/or angiographical detectable vasospasm received an intra-arterial spasmolysis.

We compared three different intra-arterial treatment strategies for preventing vasospasm-caused infarctions. The application of nimodipine only and the combination of nimodipine and milrinone were superior to milrinone alone. The proven superiority refers in particular to angiographically detectable changes and the frequency of DCI-related infarctions detected by CT.

Yindeedej et al. (12) presented the first randomized controlled trial comparing intra-arterial nimodipine application vs. standard medical treatment alone. Their study demonstrated a significant improvement in motor strength within 24 h and at discharge for an adjunct intra-arterial nimodipine infusion in cases with angiographic vasospasm. Controlled trials for endovascular rescue treatment are unlikely to be performed due to the ethical concern of withholding treatment at this critical point. Adami et al. (13) treated 88 patients with intra-arterial nimodipine infusion and in selected cases with additional percutaneous transluminal angioplasty (*n* = 18), of whom 53% developed new infarcts. These findings align with our results. Other investigators reported 21–62% of infarction rates despite intra-arterial spasmolysis with nimodipine (14). Castle-Kirsbaum et al. (5) summarized ten single-center, observational studies with 818 patients who underwent milrinone treatment. Five studies investigated the effect of primary intravenous milrinone on neurological deficits associated with DCI. Development of DCI occurred at similar rates with intravenous (291/330; 88%) and combined intra-arterial-intravenous (84/94; 89%) therapy. These concordant results question the intra-arterial monotherapy with milrinone.

Our study only focused on short-term, repetitive intra-arterial spasmolysis in the angiography suite, which is the common form. Leaving the intra-arterial microcatheter in the ICA for continuous infusion of intra-arterial nimodipine in the ICU is a less frequently practiced method (15). An indwelling microcatheter in the ICA can lead to embolism and needs close observation in the ICU. This time-consuming process is not always realizable, requiring high staff expenses. Secondary systemic distribution of the vasodilator often increases the vasopressor demand to counteract this effect peripherally (16). Weiss et al. (17) investigated the potential of induced hypertension vs. continuous intra-arterial nimodipine infusion as a rescue treatment for DCI. In the latest guidelines, induced hypertension is the recommended primary rescue therapy and intra-arterial spasmolysis is an adjunct with the weaker recommendation. In their study, induced hypertension had a limited potential to overcome hypoperfusion in DCI. Intra-arterial nimodipine with close monitoring and preemptive lowering of pressure target after its induction was suggested as a safe alternative to alleviate total norepinephrine requirements and potentially reduce complication rates.

Nimodipine and milrinone influence the amount of cytosolic calcium in a different manner. Both drugs had a highly significant vasodilating effect on the measured vessel segments in our patients, except the extradural C5 segment in nimodipine monotherapy. Nimodipine is the only FDA-approved drug that has been shown to reduce the rate of DCI and poor outcomes after SAH (1).

Reports regarding the clinical effect of Milrinone in patients with vasospasm after SAH paint a heterogenous picture (1, 5, 18). On the one hand, an immediate clinical benefit is described in the majority of reports for intra-arterial application with vascular relaxation (19, 20). On the other hand, proof of long-term outcome and clinical benefit cannot be offered (18).

PDE3 hydrolyses cyclic adenosine 3'5'-monophosphate (cAMP) and cyclic guanosine 3'5'-monophosphate (cGMP) with maximum velocity for cAMP higher than that for cGMP (21). PDE3 inhibitors have inotropic effects in the myocardium attributable to their ability to increase the accumulation of cAMP. cAMP regulates the activity of calcium channels in the sarcolemma and sarcoplasmic reticulum of cardiac myocytes and vascular smooth muscle cells. It reduces the amount of calcium available for the contraction of vascular smooth muscle cells. The increase of second messengers in cell functions may bring along a broad range of side effects (21–23). In our retrospective analysis, 1 mg milrinone provoked a self-limiting cardiac arrhythmia in one patient, similar to the case report of Vimala et al. (23). In former studies, polyuria, hypokalemia, and hyponatremia were expected adverse effects (5, 22). Myocardial ischemia and thrombocytopenia were infrequent.

In retrospective clinical studies (8, 24), the intra-arterial spasmolysis with nimodipine and/or milrinone was analyzed using diameters of the major vascular trunks without defined vessel segments for interstudy comparison. Arakawa et al. (19) published a case series for milrinone with seven patients and reported a very high mean change of the M1 vessel diameter of 76.8%. The smaller the pre-infusion diameter of the spastic segment is, the higher the post-infusion effect can be. In the study of Hejcl et al. (25) (*n* = 34), on average, the distal ICA segment dilated by 12% (*p* < 0.001), the A1 segment by 35% (*p* < 0.001) and the M1 segment by 40% (*p* < 0.001) after milrinone or nimodipine administration. Shankar et al. (20) (*n* = 14) reported a mean percentage angiographic improvement after intra-arterial Milrinone application in supraclinoid ICA of 11.32%, M1 and M2 segment of MCA of 37.5%, and A1 and A2 segment of ACA 38.69%. These results match our IPR. The discordant value of Milrinone with an IPR of 112% in the M2 segment is most probably caused by anatomical variations, rendering this measuring point especially difficult, e.g., caused by unequal distribution of M2 branches.

The delay of 5 to 7 days between SAH and the onset of CVS is not entirely consistent with the appearance of most putative spasmogens in cerebrospinal fluid after SAH (1, 26). Multiple factors are involved in the pathophysiology of vessel narrowing, e.g., structural blood-induced arteriopathy, limiting the effectiveness of spasmolytic agents (1, 27). Light and electron microscopic alterations in cerebral artery structure after SAH have been described in human postmortem and intraoperative specimens since 1964 (27). Furthermore, the contribution of leptomeningeal collaterals as one mechanism to prevent critical ischemia is under discussion (28). Already in 1969, Crompton found that the distribution of previously reported small patchy infarcts was not always correlated with the territory of the vessel bearing the aneurysm, suggesting that these infarcts cannot be explained solely by the artery's spasm. The connection of clinical results or imaging findings with the applied treatment schemes must therefore be evaluated cautiously.

### Limitations

It has to be noted that our retrospective study is focused on imaging results and does not allow a reliable conclusion from the considered intervention on the clinical outcome. Randomized controlled trials are challenging to realize due to the disease pattern's severity and infrequency. The inhomogeneous sample size per drug in our analysis does not allow for a robust statistical assessment of the effect of treatment on vessel diameter. Furthermore, unequal distribution of the Fisher score put the patients treated with the nimodipine/milrinone combination in a worse position. A prospective design is needed to refer valuable clinical outcome data to pharmacological intra-arterial CVS treatment.

Twenty-two angiograms were analyzed for monotherapy with nimodipine or milrinone even though the respective patients received the other drug in another endovascular intervention for the same reason, which nevertheless may justify this consideration since both drugs have a short half-life, being eliminated after 12 h. Nevertheless, there may result a confounder regarding possible cumulative effects.

## Conclusions

For intra-arterial pharmacological spasmolysis after SAH, nimodipine seems superior to milrinone with regard to the local effect on cerebral vessels. The combined therapy with nimodipine and milrinone prevented DCI-related infarctions equal to nimodipine monotherapy in severe CVS in our cohort. Nimodipine and milrinone may be used complementary in an escalation scheme with the administration of nimodipine first, complemented by milrinone in cases of severe CVS. Following former studies, our results question the monotherapy with milrinone for intra-arterial spasmolysis.

## Data Availability Statement

The datasets presented in this study can be found in online repositories. The name of the repository and accession number can be found at: Zenodo, https://zenodo.org/, href=https://zenodo.org/record/6374148.

## Ethics Statement

The studies involving human participants were reviewed and approved by Ethical Committee of the Leipzig University. The patients/participants provided their written informed consent to participate in this study.

## Author Contributions

UQ and SS performed the endovascular interventions. JJ was responsible for data acquisition and drafting the article. HM performed image analysis. SZ was responsible for patient monitoring. DL and KG performed neurosurgical interventions. K-TH wrote and reviewed the paper. RW revised the article critically for important intellectual content. DH performed the statistical review. SM performed vascular analysis. CR designed the study, wrote the paper, and performed interventions. All authors contributed to the article and approved the submitted version.

## Funding

The work was supported by the Open Access Publishing Fund of Leipzig University, supported by the German Research Foundation within the Open Access Publication Funding program.

## Conflict of Interest

The authors declare that the research was conducted in the absence of any commercial or financial relationships that could be construed as a potential conflict of interest.

## Publisher's Note

All claims expressed in this article are solely those of the authors and do not necessarily represent those of their affiliated organizations, or those of the publisher, the editors and the reviewers. Any product that may be evaluated in this article, or claim that may be made by its manufacturer, is not guaranteed or endorsed by the publisher.

## Supplementary Material

Supplement 1 Standard operating procedure. The internal standard operating procedure for patients suffering from aneurysmatic SAH. *CT*, computer tomography; *CTA*, computer tomography angiography; *DSA*, digital subtraction angiography; *EVD*, external ventricular drainage; *ICH*, intracerebral hemorrhage; *SAH*, subarachnoid hemorrhage; *TCD*, transcranial doppler.
