# Supplementary figures and images for: Nimodipine vs. Milrinone – Equal or Complementary Use? A Retrospective Analysis
Source: Front Neurol. 2022 Jul 14;13:939015. doi: 10.3389/fneur.2022.939015 (PMC9330364; doi:10.3389/fneur.2022.939015)

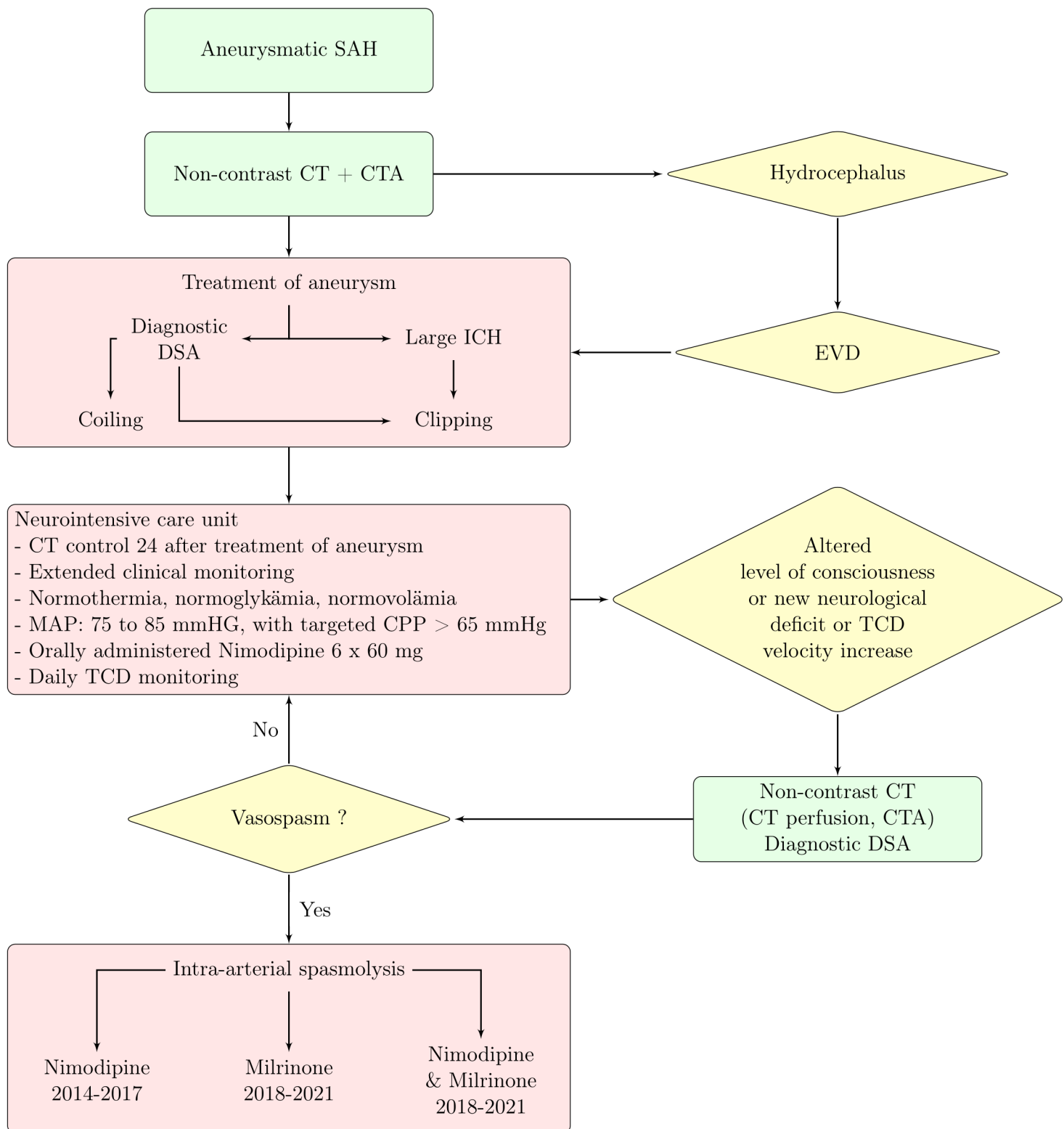

Supplement: Supplement 1 — Standard operating procedure. The internal standard operating procedure for patients suffering from aneurysmatic SAH. CT, computer tomography; CTA, computer tomography angiography; DSA, digital subtraction angiography; EVD, external ventricular drainage; ICH, intracerebral hemorrhage; SAH, subarachnoid hemorrhage; TCD, transcranial doppler. [file Data_Sheet_1.pdf]
